# Epidemiology of multiple chronic conditions: an international perspective

**DOI:** 10.15256/joc.2013.3.25

**Published:** 2013-12-24

**Authors:** François G. Schellevis

**Affiliations:** ^1^NIVEL (Netherlands Institute for Health Services Research), Utrecht, The Netherlands; Department of General Practice and Elderly Care Medicine/EMGO Institute for Health and Care Research, VU University Medical Center, Amsterdam, The Netherlands

**Keywords:** multimorbidity, multiple chronic conditions, prevalence, methodology, quality of care

## Abstract

The epidemiology of multimorbidity, or multiple chronic conditions (MCCs), is one of the research priority areas of the U.S. Department of Health and Human Services (HHS) by its Strategic Framework on MCCs. A conceptual model addressing methodological issues leading to a valid measurement of the prevalence rates of MCCs has been developed and applied in descriptive epidemiological studies. Comparing these results with those from prevalence studies performed earlier and in other countries is hampered by methodological limitations. Therefore, this paper aims to put the size and patterns of MCCs in the USA, as established within the HHS Strategic Framework on MCCs, in perspective of the findings on the prevalence of MCCs in other countries. General common trends can be observed: increasing prevalence rates with increasing age, and multimorbidity being the rule rather than the exception at old age. Most frequent combinations of chronic diseases include the most frequently occurring single chronic diseases. New descriptive epidemiological studies will probably not provide new results; therefore, future descriptive studies should focus on the prevalence rates of MCCs in subpopulations, statistical clustering of chronic conditions, and the development of the prevalence rates of MCCs over time. The finding of common trends also indicates the necessary transition to a next phase of MCC research, addressing the quality of care of patients with MCCs from an organizational perspective and with respect to the content of care.

Journal of Comorbidity 2013;3:36–40

## Introduction

The aging of mankind, which we are currently witnessing all over the world, is unprecedented in human history. Life expectancy breaks records, and a 100th birthday is no longer a rare event. The reverse side of this – in many respects successful – development is the increasing number of people with chronic diseases. The occurrence of many chronic diseases is related to increasing age, which can often be explained by long-lasting exposure to risk factors (e.g. smoking, overweight, physically burdensome labor). In 1986, Olshansky and Ault first predicted this development by announcing the entry into the “Fourth Stage of the Epidemiologic Transition: The Age of Delayed Degenerative Diseases” [1], calling for “new ways of thinking about aging, disease, morbidity, mortality, and certainly how life will be lived in advanced ages in the very near future.” However, at that time, Olshansky and Ault did not specifically predict the increasing number of people with multiple chronic conditions (MCCs), which has received increasing attention over the last few decades, and which represents a major challenge for public health, healthcare, and social care as recognized by the U.S. Department of Health and Human Services (HHS) by its Strategic Framework on Multiple Chronic Conditions [2]. Although the relevance of MCCs, or comorbidity, was demonstrated by Feinstein back in 1970 [3], the attention to MCCs has only increased in the last decade when healthcare professionals became aware that many people who visited them daily for the management of their multiple chronic diseases did not resemble the “model” patients with a single chronic disease who were included in the randomized clinical trials to establish the evidence for effective treatment and management [4]. This also signifies the end of what one could call the first wave of evidence-based medicine: effective treatments have been established for many chronic diseases; however, the effectiveness of treatment has been established in homogeneous single-disease study populations in whom the existence of other chronic conditions is usually an exclusion criterion [5].

Awareness of the relevance of MCCs initially led to the establishment of the size, patterns, and determinants of MCCs by descriptive epidemiological research. Many researchers from all over the world performed such analyses [6], often by using existing administrative or clinical databases. In addition, within the HHS Strategic Framework on MCCs, analyses have been published recently on the epidemiology of MCCs across different settings in US healthcare [7].

Insight into the size and patterns of MCCs in different settings is necessary for the development of preventive and management strategies, and for prioritizing studies to establish evidence for effective treatment of patients with MCCs. This paper aims to put the size and patterns of MCCs in the USA, as established within the HHS Strategic Framework on MCCs, in perspective of the findings on the prevalence of MCCs in other countries. This broader perspective will provide insight into the generalizability of findings at national levels.

## MCC prevalence rates

Valid comparisons of the prevalence rates of multimorbidity require a rigorous methodological approach, with unequivocal definitions, and inclusion criteria for existing data or strict rules for collection of new data. Several authors have addressed the methodological challenges in providing the MCC prevalence rates to be comparable with other studies. Criteria for comparability of the MCC prevalence rates include commonality in: (1) the definition of chronicity; (2) the level at which chronic conditions are defined (e.g. transient ischemic attack or cerebrovascular disease); (3) the number of chronic conditions under study; (4) the definition of multimorbidity; and (5) the study population, healthcare setting, or data source (e.g. administrative data, clinical databases, or population surveys) [8–10]. Within the HHS Strategic Framework on MCCs, a conceptual model has been developed which addresses most of these criteria [11].

The two most recent systematic reviews on prevalence studies on multimorbidity [8, 9] had to deal with the heterogeneity of the performed studies. The prevalence study performed within the HHS Strategic Framework on MCCs [10] applied their own conceptual model [11]. All of these studies had the definition of multimorbidity in common: two or more chronic conditions in the same person. The results of these prevalence studies are summarized in Table 1 [8–10, 12–29]. This table shows the wide variation in the prevalence rates of multimorbidity as can be expected from heterogeneous data sources. However, there is a clear and consistent trend towards higher prevalence rates at older age. Moreover, almost all studies show that at advanced age, multimorbidity is the rule rather than the exception.

## MCC patterns

Some studies [e.g. 10], and the study performed within the U.S. HHS Strategic Framework on MCCs [29], have paid attention to the pattern of MCCs, i.e. which chronic conditions often co-occur. These studies all provide the same consistent results, which are highly predictable by the prevalence rates of the most frequently occurring single chronic conditions. The five most prevalent combinations of two chronic conditions (“dyads”) usually include hypertension, (osteo)arthritis, ischemic heart disease, diabetes mellitus, with depression and cancer occurring more prominently in dyads of chronic conditions at older age. Although the comparability of studies on patterns of MCCs will highly depend on the level at which chronic conditions are defined, this does not seem to affect the patterns of dyads of chronic conditions.

## Next phase in MCC research

These global comparisons between the MCC prevalence rates established in the USA and abroad and the patterns of MCCs led to the conclusion that new descriptive epidemiological studies will probably not provide significantly different results. This does not mean that these types of studies should no longer be undertaken. There are still many valid reasons for continuing in-depth descriptive studies, e.g. in specific subpopulations, defined by sociodemographic (e.g. ethnic, socioeconomic), medical (diseases), or functional (disability) characteristics. Studies into (statistical) clustering of diseases, showing higher prevalence rates of combinations of chronic diseases than can be expected by chance, may provide clues for further exploring etiological factors [30]. Moreover, the development of MCCs over time needs to be monitored. Such studies will provide clues for preventing or delaying the occurrence of MCCs, and they will support healthcare planning by demonstrating which patients are in highest need of care.

However, we must also acknowledge that the body of knowledge about the epidemiology of MCCs is nowadays sufficiently large to take a next step by initiating studies addressing the quality of care for patients with MCCs. The U.S. HHS Strategic Framework on MCCs took up this challenge by prioritizing research into both the organization and the content of healthcare for patients with MCCs [2]. Two recently published systematic reviews from outside the USA showed that there is no evidence for any care intervention to be effective for patients with MCCs [31, 32]. This calls for further development and evaluation of, for example, case-management programs or programs involving geriatric expertise. Regarding the content of care, inclusion of patients with MCCs in clinical trials should be facilitated, parallel to the development of methodologies to deal with heterogeneity. In addition, the impossibility of developing clinical guidelines for all possible combinations of diseases should be acknowledged, leading to priorities being set. Criteria for priority setting could be the level of potential risks for interactions or contradictions between clinical guidelines for the separate chronic conditions. The development and evaluation of effective care programs for patients with MCCs represents an even bigger challenge because programs will have to be tailored to national healthcare systems regarding both the organization and content of care.

## Concluding remarks

The aim of this paper was to put the size and patterns of MCCs in the USA, as established within the HHS Strategic Framework on MCCs, into perspective of the findings on the prevalence of MCCs in other countries. Although the comparability of descriptive epidemiological studies is hampered by many methodological pitfalls, general trends can be observed in studies performed in populations all over the world: an increasing prevalence of MCCs with age, and MCCs at older age being the rule rather than the exception. Moreover, as can been expected, the highest prevalence rates of combinations of diseases is determined by the prevalence rate levels of the separate diseases. Further descriptive studies should go into more depth, especially by studying MCCs in different subpopulations. These consistent findings call for a next phase in MCC research, focusing on the quality of care for patients with MCCs, both from an organizational perspective and in terms of the content of care.

## Figures and Tables

**Table 1:** Prevalence of multiple chronic conditions (MCCs) from studies included in systematic reviews outside of the USA and a study performed within the U.S. Health and Human Services Strategic Framework on MCCs.

Source	Country	Study population, age (years)	Number of chronic conditions under study	Age (years) and prevalence of MCC
*Studies cited in Fortin et al., 2012 [8] and/or Marengoni et al., 2011 [9]*				
van den Akker et al., 1998 [12]	Netherlands	General population, all ages	335	80+: 74% (males), 80% (females)
Britt et al., 2008 [13]	Australia	General practitioner patients, all ages	18	75+: 83%
Cazale and Dimitru, 2008 [14]	Canada	General population, 12+	7	12–19: <1% 20–24: 1% 25–44: 2% 45–64: 15% 65–79: 39% 80+: 49%
Fuchs et al., 1998 [15]	Israel	General population, 75–94	14	65%
Fortin et al., 2005 [16]	Canada	Residents, 18+	All diagnoses	65+: 98%
Kadam et al., 2007 [17]	UK	Visitors of general practitioners, 50+	185	81%
Loza et al., 2009 [18]	Spain	Living in community, 20+	All diagnoses	20+: 30%
MacLeod et al., 2004 [19]	UK	General population, 18+	8	30%
Marengoni et al., 2008 [20]	Sweden	Living in community and institutions, 78+	All chronic conditions	78+: 55%
Menotti et al., 2001 [21]	Finland, Netherlands, Italy	General population, 65–84, males	7	23% (Finland)13% (Netherlands)15% (Italy)
Minas et al., 2010 [22]	Greece	Visitors of primary health care centers, 14+	All chronic diseases	54–75: 23%
Nagel et al., 2008 [23]	Germany	General population, 50–75	13	67%
Naughton et al., 2006 [24]	Ireland	General population, all ages	9	60%
Rapoport et al., 2004 [25]	Canada	General population, 20+	22	20–39: 11% 40–59: 26% 60–79: 55% 80+: 64%
Schellevis et al., 1993 [26]	Netherlands	General population, all ages	5	<65: 0.3% 65+: 3.6%
Schram et al., 2008 [10]	Netherlands	General population, 55+	Depending on setting: 12–13 (population setting); 68–83 (listed patients in general practices)	55+: 56–72%
Uijen and van de Lisdonk, 2008 [27]	Netherlands	General population, all ages	All chronic diseases	65–74: 30%75+: 55%
Walker, 2007 [28]	Australia	General population, 20+	All long-lasting conditions	70+: 85%
*Ward et al., 2013 [29]*	USA	General population, 18+	10	18–44: 7% (males); 9% (females) 45–64: 33% (males); 35% (females) 65+: 63% (males); 62% (females)
